# Antiferromagnetic Phase Induced by Nitrogen Doping in 2D Cr_2_S_3_

**DOI:** 10.3390/ma15051716

**Published:** 2022-02-24

**Authors:** Wenda Zhou, Mingyue Chen, Cailei Yuan, He Huang, Jingyan Zhang, Yanfei Wu, Xinqi Zheng, Jianxin Shen, Guyue Wang, Shouguo Wang, Baogen Shen

**Affiliations:** 1 School of Materials Science and Engineering, Beijing Advanced Innovation Center for Materials Genome Engineering, University of Science and Technology Beijing, Beijing 100083, China; wdzhou@xs.ustb.edu.cn (W.Z.); mychen@xs.ustb.edu.cn (M.C.); hhuang@ustb.edu.cn (H.H.); jyzhang@ustb.edu.cn (J.Z.); yanfeiwu@ustb.edu.cn (Y.W.); zhengxq@ustb.edu.cn (X.Z.); jxshen@ustb.edu.cn (J.S.); gywang@xs.ustb.edu.cn (G.W.); 2 Jiangxi Key Laboratory of Nanomaterials and Sensors, School of Physics, Communication and Electronics, Jiangxi Normal University, Nanchang 330022, China; 3 Beijing National Laboratory for Condensed Matter Physics, Institute of Physics, Chinese Academy of Sciences & University of Chinese Academy of Sciences, Beijing 100190, China

**Keywords:** 2D magnetic materials, in situ nitrogen doping, antiferromagnetism

## Abstract

Exploration for the new members of air-stable 2D antiferromagnetic magnets to widen the magnetic families has drawn great attention due to its potential applications in spintronic devices. In addition to seeking the intrinsic antiferromagnets, externally introducing antiferromagnetic ordering in existing 2D materials, such as structural regulation and phase engineering, may be a promising way to modulate antiferromagnetism in the 2D limit. In this work, the in situ nitrogen doping growth of ultrathin 2D Cr_2_S_3_ nanoflakes has been achieved. Antiferromagnetic ordering in 2D Cr_2_S_3_ nanoflakes can be triggered by nitrogen doping induced new phase (space group P$\bar{3}$1c). This work provides a new route to realize antiferromagnetism in atomically thin 2D magnets and greatly extend applications of 2D magnets in valleytronics and spintronics.

## 1. Introduction

As the scaling process of traditional ferromagnet-based spin device reaches the end, interest has been fueled in the development of antiferromagnetic spintronics [1,2,3]. In contrast to ferromagnetic materials, antiferromagnets have exhibited many distinct advantages for information manipulation and storage including robust antiferromagnetic order, absence of stray fields, and teraHertz switching frequencies [4,5,6]. Recently, accompanied by the discovery of two-dimensional (2D) van der Waals (vdW) magnetic materials such as the ferromagnetic Cr_2_Ge_2_Te_6_ insulator, ferromagnetic Fe_3_GeTe_2_ metal, and intralayer-ferromagnetic (interlayer-antiferromagnetic) CrI_3_ semiconductor [7,8,9], the 2D antiferromagnet has attracted tremendous attention for exploring ultrathin antiferromagnetic spintronic devices with advanced functionalities [10,11,12]. Unfortunately, the practical application of 2D antiferromagnets is greatly limited by their air-instabilities, low ordering temperatures, and low-yield productions by exfoliation [9,13,14]. Therefore, air-stable atomically thin antiferromagnets with high-yield production are highly demanded. Except for exploring new air-stable intrinsic antiferromagnets, externally introducing antiferromagnetic ordering in existing 2D materials, such as structural regulation by virtue of the strong correlation between structural phase transition and magnetic phase transition, would be another promising strategy to modulate antiferromagnetism in the 2D limit and widen the magnetic materials systems.

In this paper, we firstly realized in situ nitrogen doping growth of 2D Cr_2_S_3_ nanoflakes successfully by a plasma-enhanced chemical vapor deposition (PECVD) system under N_2_ atmosphere. X-ray diffraction (XRD) patterns at variable temperatures demonstrated that in situ nitrogen doping could induce a new phase (space group P$\bar{3}$1c), which was not observed in previously reported 2D Cr_2_S_3_ nanoflakes [15,16,17,18]. Theoretical calculations revealed the tendency of 2D Cr_2_S_3_ nanoflakes with space group P$\bar{3}$1c to exhibit an antiferromagnetic order. An anomalous peak located ~150 K was observed in the temperature dependence of magnetization (M–T curve), where a different loop shape compared with the P-Cr_2_S_3_ counterpart can be seen from M–H loops in nitrogen-doped Cr_2_S_3_ (N-Cr_2_S_3_) nanoflakes. This attractive magnetic behavior can be attributed to the competition between ferromagnetic and antiferromagnetic phases in 2D N-Cr_2_S_3_ nanoflakes. Our work may provide a new approach to manipulate antiferromagnetism in atomically thin 2D magnets and greatly extend their applications of 2D magnets in valleytronics and spintronics.

## 2. Materials and Methods

The morphologies of Cr_2_S_3_ nanoflakes were examined using Atomic Force Microscopy (AFM, Park system XE7, Suwon, South Korea). Transmission electron microscope (TEM) characterization was carried out on a FEI Talos f200x microscope (Thermo Fisher, San Francisco, CA, USA) operated at 200 KV. The X-ray photoelectron spectroscopy (XPS) spectra were recorded employing Al Kα radiation from a XSAM800 spectrometer (Kratos, Manchester, UK). The binding energy of samples was calibrated by C 1s peak. XRD patterns at variable temperatures were examined using Rigaku SmartLab (Tokyo, Japan). The magnetic properties were studied by a physical properties measurement system (PPMS, Quantum Design, San Diego, CA, USA) with a vibrating sample magnetometer option (Ever Cool II).

## 3. Results and Discussion

**Figure 1:**
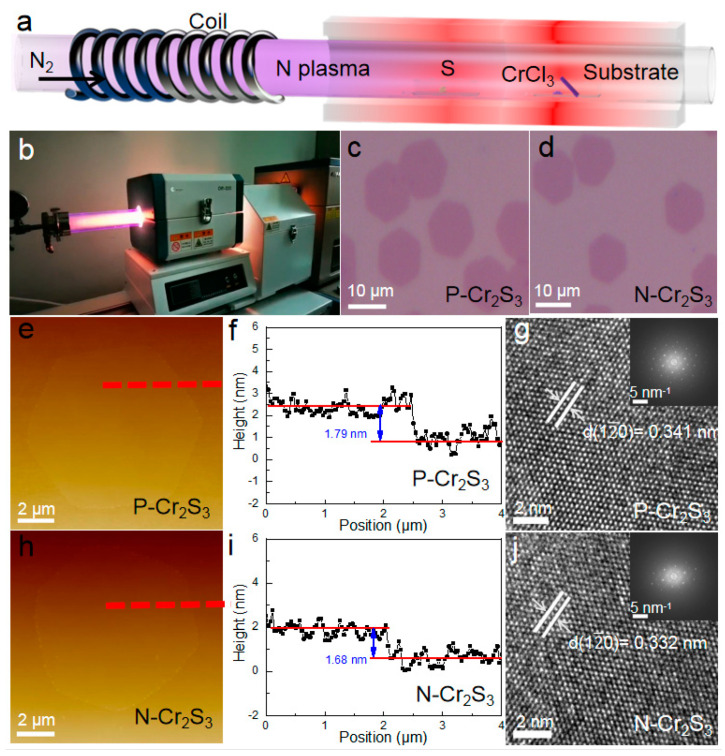
(**a**) Synthesis diagram of Cr_2_S_3_ nanoflakes. (**b**) Image of PECVD system. Optical microscope images of (**c**) P-Cr_2_S_3_ and (**d**) N-Cr_2_S_3_ on SiO_2_/Si substrate, respectively. (**e**) and (**h**) AFM topographic images, (**f**) and (**i**) corresponding height profiles, (**g**) and (**j**) typical HRTEM of P-Cr_2_S_3_ and N-Cr_2_S_3_ nanoflake, respectively. Inset of (**g**) and (**j**): the corresponding FFT patterns.

Ultrathin N-Cr_2_S_3_ nanoflakes were successfully synthesized by a PECVD system. The growth configuration of N-Cr_2_S_3_ nanoflakes was depicted in Figure 1a. S powder (99.9%, Aladdin, China) precursor was placed in the center of low temperature zone. A powder mixed with CrCl_3_ (99.9%, anhydrous, Alfa Aesar, Tewksbury, USA) and NaCl (99.5%, Aladdin, China) with a weight ratio of 10:1 was loaded into quartz boat in the middle of high temperature zone. The SiO_2_/Si growth substrate (facing down) was slanted downward into the chute (~45°) of the quartz boat. The substrate tilted 45° with respect to the carrier gas flow may reduce the downstream depletion of precursors, which facilitates the nanoflake growth. The S and CrCl_3_ powders were heated up concurrently to 240 and 760 °C at a N_2_ flow rate of 60 sccm. A radio frequency plasma reactor (CHY, China, input power 100 W) was turned on to generate nitrogen plasma, as shown in Figure 1b. After 15 minutes, the plasma reactor was turned off and the PECVD system was cooled down naturally to room temperature under N_2_ atmosphere. The growth conditions of pristine Cr_2_S_3_ (P-Cr_2_S_3_) nanoflakes were kept as same as those of N-Cr_2_S_3_ nanoflakes, except without turning on the plasma reactor. Moreover, to control the level of nitrogen doping in Cr_2_S_3_ nanoflakes, by adjusting the input power (50 W) of plasma reactor, low-density nitrogen-doped Cr_2_S_3_ nanoflakes can be synthesized (see Figures S1 and S2, Supporting Information). However, with too high input power of the plasma reactor, Cr_2_S_3_ nanoflakes cannot be produced (see Figure S3, Supporting Information).

Figure 1c,d show optical microscope images of P-Cr_2_S_3_ and N-Cr_2_S_3_ nanoflakes grown on SiO_2_/Si substrate, respectively, indicating a hexagonal shape with an average size of around 10 micrometers. These typical microscopic images confirmed that no obvious morphological change was observed in P-Cr_2_S_3_ nanoflakes after nitrogen plasma doping. Furthermore, the thickness of P-Cr_2_S_3_ and N-Cr_2_S_3_ nanoflakes on SiO_2_/Si substrate was determined to be ~1.79 and ~1.68 nm, respectively, by AFM shown in Figure 1e,f,h,i. Above results indicate that we have fulfilled the one-unit-cell thickness synthesis of Cr_2_S_3_ nanoflakes [18]. Moreover, a little thickness difference between P-Cr_2_S_3_ and N-Cr_2_S_3_ nanoflakes may be due to the smaller atomic size of N atoms compared with S atoms [19]. To further resolve the crystallinity and detailed lattice structure of the as-grown Cr_2_S_3_ nanoflakes, TEM characterization was performed. A typical high-resolution TEM (HRTEM) image of the P-Cr_2_S_3_ was shown in Figure 1g, and the interplanar spacing was measured to be 0.341 nm referring to the (120) plane, in good agreement with previous reports [17]. It is worth noting that there is a slight difference between the interplanar spacing of N-Cr_2_S_3_ (0.332 nm) and P-Cr_2_S_3_, which is mainly due to the incorporation of nitrogen into the one-unit-cell Cr_2_S_3_ nanoflakes, which is well supported by AFM results [19]. More importantly, no defects or grain boundaries were observed, revealing the uniformity and high quality characteristics of P-Cr_2_S_3_ and N-Cr_2_S_3_ samples. Such high-crystal-quality features are consistent with the fast Fourier transform (FFT) mode, with a single set of hexagonal diffraction spots (inset of Figure 1g,j). The AFM and TEM observations demonstrate that the nitrogen plasma doping has no significant effect on the surface morphology and crystallinity of one-unit-cell Cr_2_S_3_ nanoflakes.

**Figure 2:**
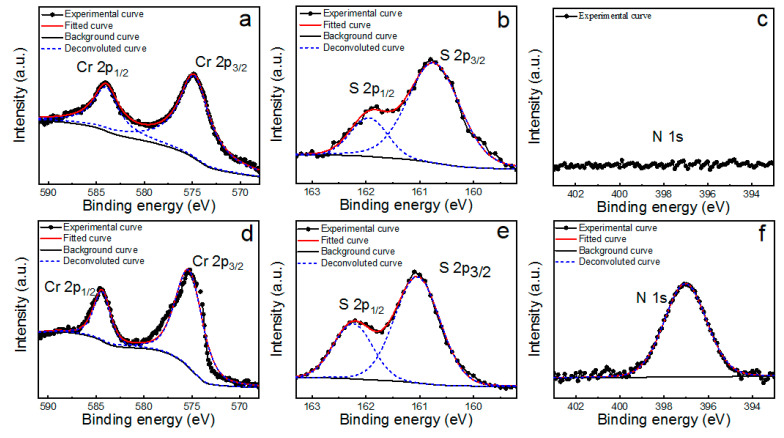
XPS spectra of (**a**) and (**d**) Cr 2p, (**b**) and (**e**) S 2p, (**c**) and (**f**) N 1s of P-Cr_2_S_3_ and N-Cr_2_S_3_ nanoflakes, respectively.

The elemental composition and bonding state of the as-grown Cr_2_S_3_ nanoflakes were identified by XPS (Figure 2). As manifested in Figure 2a, two strong peaks at about 574.8 and 584.1 eV can be clearly observed in Cr 2p spectra of P-Cr_2_S_3_ nanoflakes, which are ascribed to the doublet Cr 2p_3/2_ and Cr 2p_1/2_, respectively. The peaks of S 2p_3/2_ and S 2p_1/2_ orbits corresponding to divalent sulfide ions (S^2−^) located at about 160.8 and 161.9 eV (Figure 2b). These featuring peaks are in good agreement with the reported results of Cr_2_S_3_ nanoflakes synthesized by CVD method [15,17]. For N-Cr_2_S_3_ nanoflakes shown in Figure 2d,e, the 2p_3/2_ and 2p_1/2_ states of Cr were confirmed by the peaks at about 575.2 and 584.4 eV, and the S 2p_3/2_ and S 2p_1/2_ states at about 161.1 and 162.3 eV, respectively. In comparison with P-Cr_2_S_3_, the binding energies of Cr 2p and S 2p regions of N-Cr_2_S_3_ exhibit a negative shift of about 0.3 eV in the XPS spectra. This negative shift indicates the Fermi level moves to the maximum of valence band, a characteristic feature of p-type doping caused by nitrogen doping, which is well consistent with previous reports [19]. For N 1s spectra, in contrast to the P-Cr_2_S_3_, an emerging peak at 397.1 eV was observed in N-Cr_2_S_3_, which was attributed to Cr-N bonds in CrN (397.0 eV) as reported previously [20], demonstrating the successful doping of nitrogen into P-Cr_2_S_3_. Furthermore, no clear N peak was observed in the S 2p region, suggesting that there was no reaction between sulfur and nitrogen. Additionally, the atomic ratio of S:Cr is determined according to the equation $\frac{C_{S}}{C_{\mathrm{Cr}}}=\frac{\left(I_{S}{/\mathrm{ASF}}_{S}\right)}{\left(I_{\mathrm{Cr}}{/\mathrm{ASF}}_{\mathrm{Cr}}\right)}$ [19,21], where *ASF_S_* and *I_S_* are the atomic sensitivity factor and integrated areas of S 2p_3/2_, respectively. Similarly, *ASF_Cr_* and *I_Cr_* are the atomic sensitivity factor and integrated areas of Cr 2p_3/2_, respectively. According to the XPS spectra of P-Cr_2_S_3_ and N-Cr_2_S_3_, the atomic ratio of S:Cr is estimated to be 1.52:1 and 1.33:1, respectively, further indicating the replacement of sulfur atoms by nitrogen atoms in P-Cr_2_S_3_.

**Figure 3:**
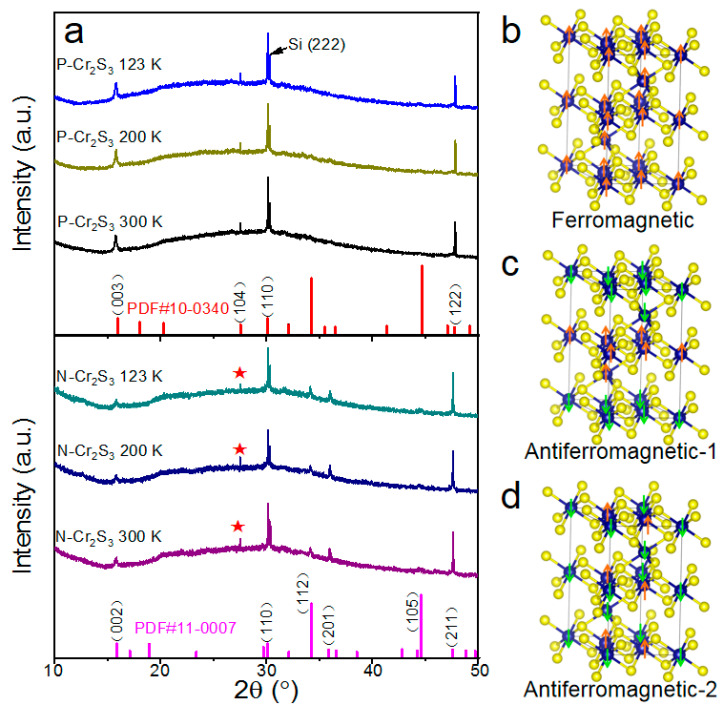
(**a**) XRD patterns at variable temperatures for P-Cr_2_S_3_ (**top**) and N-Cr_2_S_3_ (**bottom**) nanoflakes. Diffraction peaks marked with stars in N-Cr_2_S_3_ indicate rhombohedral Cr_2_S_3_ (PDF #10-0340) with space group (R$\bar{3}$) still exist in N-Cr_2_S_3_. (**b**) Schematic structure of N-Cr_2_S_3_ with ferromagnetic configuration. (**c**,**d**) Schematic structures of N-Cr_2_S_3_ with two possible antiferromagnetic configurations. Orange and green arrows denote opposite spin orientations.

To clarify the crystal structure of as-grown Cr_2_S_3_ nanoflakes, XRD patterns at various temperatures were collected. As shown in Figure 3a (top), the XRD peaks of P-Cr_2_S_3_ at 300 K can be well indexed by the standard rhombohedral Cr_2_S_3_ (PDF #10-0340) with space group (R$\bar{3}$) [16]. Interestingly, as manifested in Figure 3a (bottom), the XRD peaks of N-Cr_2_S_3_ at 300 K can be indexed by two sets of standard rhombohedral Cr_2_S_3_ structures including PDF #10-0340 and PDF #11-0007, indicting the coexistence of space group R$\bar{3}$ and space group P$\bar{3}$1c in N-Cr_2_S_3_ nanoflakes. Those results demonstrate that in situ nitrogen doping could induce a structural phase transition in 2D Cr_2_S_3_ nanoflakes. Moreover, there is no obvious change in XRD patterns of P-Cr_2_S_3_ and N-Cr_2_S_3_ with the temperature variation (from 123 K to 300 K), respectively. 

Based on XRD results, theoretical calculations were performed to explore the magnetic properties of the new phase (space group P$\bar{3}$1c) of N-Cr_2_S_3_. All the spin theoretical simulations in this work were conducted on a Vienna ab initio Simulation Package (VASP) [22]. The electron–electron exchange and correlation interactions were evaluated by the generalized gradient approximation (GGA) with the Perdew–Burke–Emzerhof (PBE) functional form, while the core-electron (valence electron) interactions were represented by implanted the projector augmented-wave (PAW) methods [23]. Plane-Wave basis function was set with a kinetic cut-off energy of 550 eV. Relaxing the force below 0.02 eV/Å was used to optimize the ground-state atomic geometries, and the convergence criteria for energy set as 1.0 × 10^−5^ eV/cell. By using a Monkhorst–Pack meshes with 4 × 4 × 2, the Brillouin zone was sampled. Electronic structures, total energy of models, and stress or force relaxations were calculated by the Gaussian method. In order to better depict the intermolecular interaction, DFT-D3 method of Grimme was employed to describe van der Waal (vdw) interactions [24]. The equation is E_bonding energy_ = (E_total_ − N_Cr_u_Cr_ − N_S_u_S_)/(N_Cr_ + N_S_). In this work, the bonding energy was calculated to evaluate the stability of our structure with different magnetic configurations. Various possible magnetic configurations of N-Cr_2_S_3_ with space group P$\bar{3}$1c were investigated, including ferromagnetic (Figure 3b) and two types of antiferromagnetic configurations, as shown in Figure 3c,d. The Antiferromagnetic-1 (−0.83 eV) would be the most stable configuration due to its lowest bonding energy, compared to Ferromagnetic (−0.80 eV) and Antiferromagnetic-2 (−0.81 eV). Thus, our calculations indicate the tendency of N-Cr_2_S_3_ with space group P$\bar{3}$1c to exhibit antiferromagnetic order. Based on XRD data and theoretical calculations, a new phase with an antiferromagnetic order could be induced in 2D Cr_2_S_3_ by nitrogen doping.

**Figure 4:**
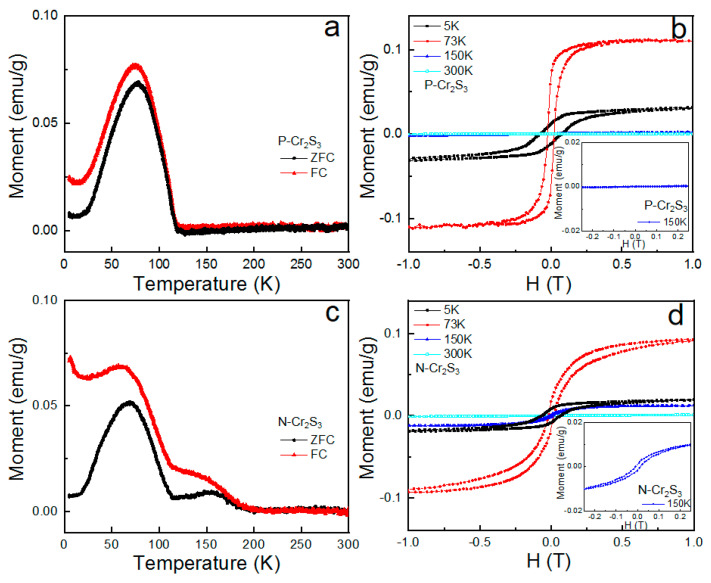
(**a**,**c**) Temperature–dependent magnetization, (**b**,**d**) magnetic hysteresis loop measurements at different temperatures for P-Cr_2_S_3_ and N-Cr_2_S_3_ nanoflakes, respectively. The inset of (**b**) and (**d**) are the corresponding magnified images of M–H curve at 150 K.

In order to check the prediction by theoretical calculations, magnetic properties of as-synthesized Cr_2_S_3_ nanoflakes were studied in a PPMS with a vibrating sample magnetometer under a parallel magnetic field due to its in-plane magnetic easy axis [17,18]. As shown in Figure 4a, zero-field-cooled (ZFC) and field-cooled (FC) magnetization curves exhibit that P-Cr_2_S_3_ nanoflakes have an obvious ferromagnetic behavior with the Curie temperature T_C_ ~120 K and a maximum magnetic susceptibility at ~73 K. The upturn of magnetization below 20 K maybe attribute to the interaction of magnetic sublattices [25]. As shown in Figure 4b, obvious hysteresis can be found in M–H curves of P-Cr_2_S_3_ nanoflakes with the temperature below 120 K (T_C_), and the hysteresis vanished when the temperature was increased above T_C_, indicating the emergence of paramagnetic property. These results are well consistent with the previous results for CVD-derived Cr_2_S_3_ nanoflakes [17,18]. For N-Cr_2_S_3_ nanoflakes, an anomalous peak at ~150 K can be clearly observed in M–T curves (see Figure 4c). It is deduced that this anomaly of temperature can be attributed to the emerging structural phase (space group P$\bar{3}$1c) of N-Cr_2_S_3_ nanoflakes. Moreover, the irreversibility behavior observed in M–T curves of N-Cr_2_S_3_ may come from domain wall pinning effect, spin reorientation phase transition or glassy state [26]. As manifested in Figure 4d, the M–H curve of N-Cr_2_S_3_ nanoflakes at ~73 K exhibits different loop shape compared with the P-Cr_2_S_3_ counterpart, with magnetic hysteresis expanding under higher applied field while pinching under lower applied field, which indicates the coexistence of ferromagnetic and canted antiferromagnetic ordering [27]. Additionally, low-density nitrogen doping in Cr_2_S_3_ nanoflakes cannot trigger the new phase (proved by XRD results, Figure S2), which exhibit similar magnetic behaviors with P-Cr_2_S_3_ nanoflakes (Figure S4). Therefore, magnetic ground states with an increase of temperature in N-Cr_2_S_3_ can be defined as coexistence of ferromagnetic and antiferromagnetic (below ~ 120 K), antiferromagnetic (120–150 K), and paramagnetic (150–300 K). These attractive magnetic behaviors prove that antiferromagnetic phase in 2D Cr_2_S_3_ nanoflakes can be triggered by an in situ nitrogen doping-induced new phase (space group P$\bar{3}$1c), which is in line with our theoretical calculations.

## 4. Conclusions

In summary, based on the large-scale synthesis of air-stable 2D ferromagnetic Cr_2_S_3_ nanoflakes, we successfully realized in situ nitrogen doping by PECVD. Variable temperature XRD results demonstrated that in situ nitrogen doping could induce a new phase in 2D N-Cr_2_S_3_ nanoflakes. Theoretical calculations and magnetic measurements prove the achievement of antiferromagnetic ordering in Cr_2_S_3_ by nitrogen doping induced a new phase (space group P$\bar{3}$1c). Our work opens up a new route for the manipulation of antiferromagnetism in the 2D limit and widens the magnetic materials systems.

## Data Availability

Data sharing is not applicable for this paper.

## Supplementary Materials

The following supporting information can be downloaded at: https://www.mdpi.com/article/10.3390/ma15051716/s1, Figure S1 XPS spectra of (a) Cr 2p, (b) S 2p, (c) N 1s of low-density nitrogen doped Cr2S3 nanoflakes.; Figure S2 XRD pattern for low-density nitrogen doped Cr2S3 nanoflakes.; Figure S3 Optical microscope image of N-Cr2S3 nanoflakes with high input power of plasma reactor (120 W).; Figure S4 (a) M-T and (b) M-H curves at different temperatures for low-density nitrogen doped Cr2S3 nanoflakes, respectively. The inset of (b) is the corresponding magnified image of M-H curve at 150 K.
