# Supplementary material for: Antiferromagnetic Phase Induced by Nitrogen Doping in 2D Cr2S3
Source: Materials (Basel). 2022 Feb 24;15(5):1716. doi: 10.3390/ma15051716 (PMC8911375; doi:10.3390/ma15051716)
Supplement: Supplementary file 1 [file materials-15-01716-s001.zip › materials-1584117-supplementary.pdf]

materials

Supplementary Materials

Antiferromagnetic Phase Induced by Nitrogen Doping in 2D Cr_2S_3

Wenda Zhou [1,2], Mingyue Chen [1,2], Cailei Yuan [2,*], He Huang [1], Jingyan Zhang [1], Yanfei Wu [1], Xinqi Zheng [1], Jianxin Shen [1], Guyue Wang [1], Shouguo Wang [1,*] and Baogen Shen [1,3,*]

[1] School of Materials Science and Engineering, Beijing Advanced Innovation Center for Materials Genome Engineering, University of Science and Technology Beijing, Beijing 100083, China; wdzhou@xs.ustb.edu.cn (W.Z.); mychen@xs.ustb.edu.cn (M.C.); hhuang@ustb.edu.cn (H.H.); jyzhang@ustb.edu.cn (J.Z.); yanfeiwu@ustb.edu.cn (Y.W.); zhengxq@ustb.edu.cn (X.Z.); jxshen@ustb.edu.cn (J.S.); gywang@xs.ustb.edu.cn (G.W.)
[2] Jiangxi Key Laboratory of Nanomaterials and Sensors, School of Physics, Communication and Electronics, Jiangxi Normal University, Nanchang 330022, Jiangxi, China
[3] Beijing National Laboratory for Condensed Matter Physics, Institute of Physics, Chinese Academy of Sciences, University of Chinese Academy of Sciences, Beijing 100190, China
* Correspondence: clyuan@jxnu.edu.cn (C.Y.); sgwang@ustb.edu.cn (S.W.); shenbg@iphy.ac.cn (B.S.)

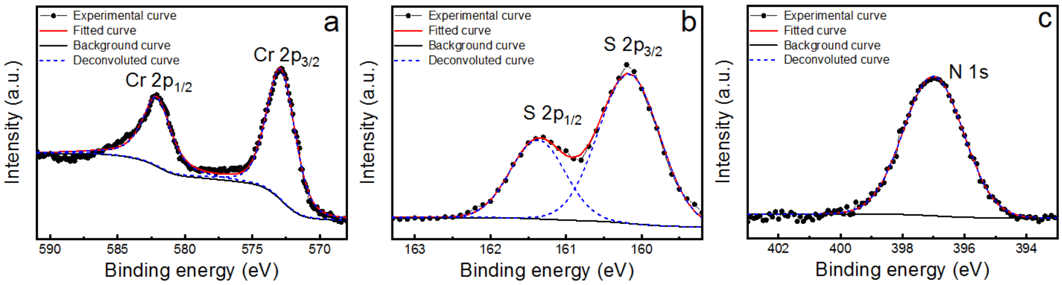

Figure S1. XPS spectra of (a) Cr 2p, (b) S 2p, (c) N 1s of low-density nitrogen doped Cr_2S_3 nanoflakes.

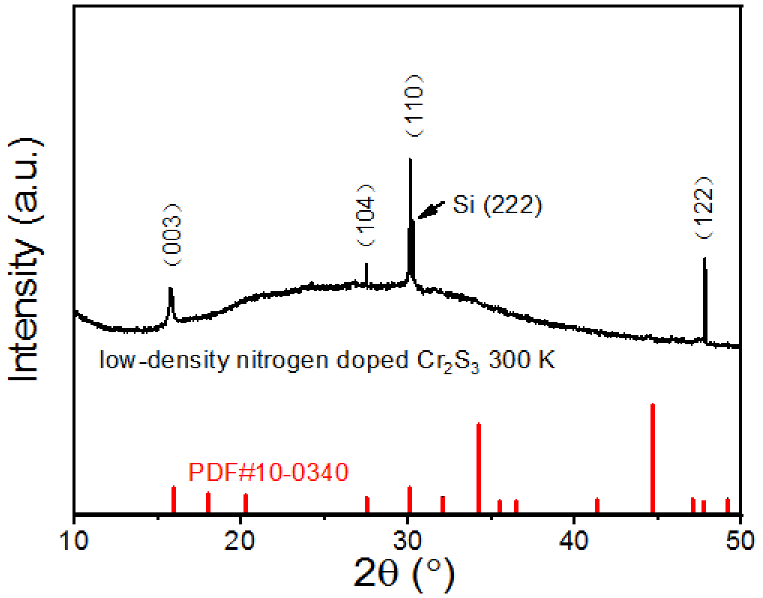

Figure S2. XRD pattern for low-density nitrogen doped Cr_2S_3 nanoflakes.

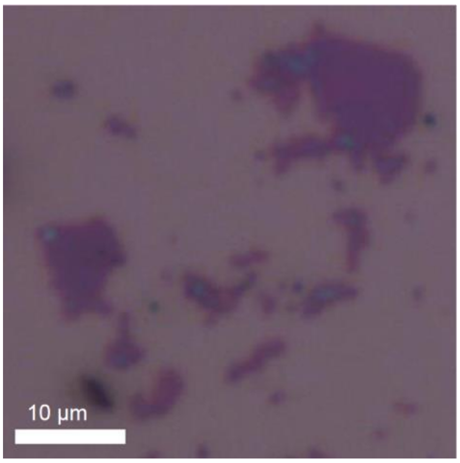

Figure S3. Optical microscope image of N-Cr_2S_3 nanoflakes with high input power of plasma reactor (120 W).

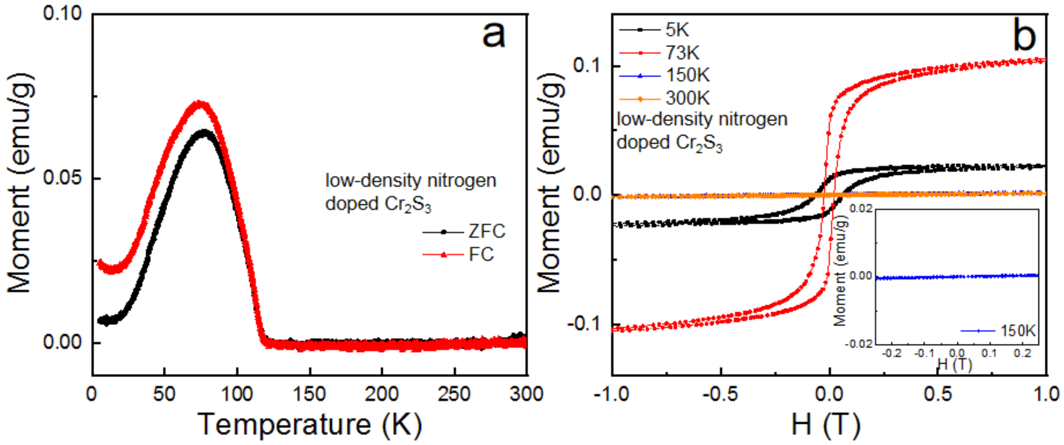

Figure S4. (a) M-T and (b) M-H curves at different temperatures for low-density nitrogen doped Cr_2S_3 nanoflakes, respectively. The inset of (b) is the corresponding magnified image of M-H curve at 150 K.
